# Cost-effectiveness of high flow nasal cannula therapy versus continuous positive airway pressure for non-invasive respiratory support in paediatric critical care

**DOI:** 10.1186/s13054-024-05148-y

**Published:** 2024-11-25

**Authors:** Zia Sadique, Silvia Moler Zapata, Richard Grieve, Alvin Richards-Belle, Izabella Lawson, Robert Darnell, Julie Lester, Kevin P. Morris, Lyvonne N. Tume, Peter J. Davis, Mark J. Peters, Richard G. Feltbower, Paul R. Mouncey, David A. Harrison, Kathryn M. Rowan, Padmanabhan Ramnarayan, the FIRST-ABC RCT Investigators, the Paediatric Critical Care Society Study Group

**Affiliations:** 1 Health Economics, Department of Health Services Research and Policy, Public Health and Policy Faculty, London School of Hygiene and Tropical Medicine, London, UK; 2 Clinical Trials Unit, Intensive Care National Audit & Research Centre, London, UK; 3 Cancer Epidemiology Unit, Nuffield Department of Population Health, University of Oxford, Oxford, UK; 4 Parent Representative, Sussex, UK; 5 Birmingham Children’s Hospital, Birmingham Women’s and Children’s NHS Foundation Trust, Birmingham, UK; 6 Institute of Applied Health Research, University of Birmingham, Birmingham, UK; 7 Faculty of Health Social Care & Medicine, Edge Hill University, Lancashire, UK; 8 Paediatric Intensive Care Unit, Bristol Royal Hospital for Children, University Hospitals Bristol and Weston NHS Foundation Trust, Bristol, UK; 9 Paediatric Intensive Care Unit, Great Ormond Street Hospital for Children NHS Foundation Trust and NIHR Biomedical Research Centre, London, UK; 10 University College London Great Ormond St Institute of Child Health, London, UK; 11 Leeds Institute for Data Analytics, School of Medicine, University of Leeds, Leeds, UK; 12 Section of Anaesthetics, Pain Medicine and Intensive Care, Department of Surgery and Cancer, Faculty of Medicine, Imperial College London, London, UK; 13 Children’s Acute Transport Service, Great Ormond Street Hospital for Children NHS Foundation Trust, London, UK

**Keywords:** High-flow nasal cannula, Continuous positive airway pressure, Paediatric critical care, Cost-effectiveness, Incremental net benefit, Non-invasive respiratory support

## Abstract

**Background:**

High flow nasal cannula therapy (HFNC) and continuous positive airway pressure (CPAP) are two widely used modes of non-invasive respiratory support in paediatric critical care units. The FIRST-ABC randomised controlled trials (RCTs) evaluated the clinical and cost-effectiveness of HFNC compared with CPAP in two distinct critical care populations: acutely ill children (‘step-up’ RCT) and extubated children (‘step-down’ RCT). Clinical effectiveness findings (time to liberation from all forms of respiratory support) showed that HFNC was non-inferior to CPAP in the step-up RCT, but failed to meet non-inferiority criteria in the step-down RCT. This study evaluates the cost-effectiveness of HFNC versus CPAP.

**Methods:**

All-cause mortality, health-related Quality of Life (HrQoL), and costs up to six months were reported using FIRST-ABC RCTs data. HrQoL was measured with the age-appropriate Paediatric Quality of Life Generic Core Scales questionnaire and mapped onto the Child Health Utility 9D index score at six months. Quality-Adjusted Life Years (QALYs) were estimated by combining HrQoL with mortality. Costs at six months were calculated by measuring and valuing healthcare resources used in paediatric critical care units, general medical wards and wider health service. The cost-effectiveness analysis used regression methods to report the cost-effectiveness of HFNC versus CPAP at six months and summarised the uncertainties around the incremental cost-effectiveness results.

**Results:**

In both RCTs, the incremental QALYs at six months were similar between the randomised groups. The estimated incremental cost at six months was − £4565 (95% CI − £11,499 to £2368) and − £5702 (95% CI − £11,328 to − £75) for step-down and step-up RCT, respectively. The incremental net benefits of HFNC versus CPAP in step-down RCT and step-up RCT were £4388 (95% CI − £2551 to £11,327) and £5628 (95% CI − £8 to £11,264) respectively. The cost-effectiveness results were surrounded by considerable uncertainties. The results were similar across most pre-specified subgroups, and the base case results were robust to alternative assumptions.

**Conclusions:**

HFNC compared to CPAP as non-invasive respiratory support for critically-ill children in paediatric critical care units reduces mean costs and is relatively cost-effective overall and for key subgroups, although there is considerable statistical uncertainty surrounding this result.

**Supplementary Information:**

The online version contains supplementary material available at 10.1186/s13054-024-05148-y.

## Background

Modes of non-invasive respiratory support (NRS), such as high flow nasal cannula therapy (HFNC) and continuous positive airway pressure (CPAP), are widely used in paediatric critical care, both in acutely ill children and those extubated after a spell of invasive ventilation [1–4]. Despite this, there is a paucity of randomised controlled trial (RCT) evidence regarding the comparative effectiveness of HFNC and CPAP, resulting in considerable variation in clinical practice [5]. In recent years, HFNC has become a preferred mode of NRS since healthcare professionals as well as caregivers perceive it to be more comfortable for children and easier to use without the need for close monitoring and specialist nursing input as required for CPAP [4, 6]. HFNC has been shown in physiological studies to improve oxygenation and work of breathing through diverse mechanisms such as nasopharyngeal washout of dead space and a degree of positive end-expiratory pharyngeal pressure (PEEP) [7, 8], while CPAP has been shown to provide higher levels of PEEP if a leak-free interface seal can be achieved [9].

The FIRST-line support for Assistance in Breathing in Children (FIRST-ABC) was a master protocol of two pragmatic RCTs, with shared infrastructure and integrated health economic evaluation, to evaluate the clinical and cost-effectiveness of HFNC compared with CPAP in two distinct critical care populations: acutely ill children (‘step-up’ RCT) and extubated children (‘step-down’ RCT) [10]. Clinical effectiveness findings from FIRST-ABC showed that, in the step-up RCT, HFNC was non-inferior to CPAP in terms of the time to liberation from all forms of respiratory support (invasive and non-invasive), whereas in the step-down RCT, HFNC did not meet the criterion for non-inferiority [11, 12]. Intubation within 48 h of randomisation was similar between the two interventions in both trials.

One of the key perceived advantages of HFNC is the ability to discharge children receiving HFNC earlier from critical care units to general wards. This provides the potential for more efficient use of scarce critical care beds and a reduction in bed-day costs. On the other hand, CPAP may be more effective, leading to more rapid patient improvement and earlier discharge from the hospital, and reducing overall costs. There is however limited evidence regarding the relative cost-effectiveness of HFNC versus CPAP in critically ill children. In one study of a decision analytic model simulating two options for treating children with bronchiolitis, HFNC care on the ward was found to be more cost-effective than providing HFNC care in a critical care unit [13]. There is also limited evidence on the relative impact of the CPAP versus HFNC on health economic outcomes such as health-related quality of life in critically ill children.

Robust data regarding cost-effectiveness is crucial to guide clinical practice and health policy regarding the choice of NRS in a critical care setting. Cost-effectiveness, in addition to clinical effectiveness, is a key part of decision-making regarding the choice of NRS; however, the paucity of this information from large multicentre RCTs so far has hampered progress. The aim of this paper is to report the pre-specified cost-effectiveness of HFNC and CPAP in the FIRST-ABC RCTs, two large multicentre pragmatic clinical trials examining the use of NRS in a paediatric critical care population, at six months following randomisation.

## Methods

### Overview

This study performs a full cost-effectiveness analysis (CEA) for each FIRST-ABC RCT (step-up and step-down) to assess the relative cost-effectiveness of HFNC versus CPAP over six months after randomisation. The CEA used patient-level healthcare resource use and outcome data collected from multiple linked data sources. Information was collated from data collected through the trial case report forms (CRFs), records from routine databases (Paediatric Intensive Care Audit Network [PICANet] and NHS Digital’s Hospital Episode Statistics [HES]), information obtained from follow-up questionnaires (health services questionnaire (HSQ) and follow-up questionnaire for health-related quality of life (HrQoL)), and information collected through local investigators. The FIRST-ABC HSQ was developed as a part of the trial to assess outpatient and community health services and resource use at six months, and was sent to each consented participant’s parents by post. Survival status at six months, including date of death for non-surviving patients, were obtained by local investigators at participating hospitals using the NHS Spine. The base case CEA followed the modified intention-to-treat principle (mITT) as per the clinical effectiveness analysis of primary outcome of FIRST-ABC RCT and reported the cost-effectiveness of HFNC versus CPAP, overall and for pre-specified subgroups. The mITT population consists of intention to treat (ITT) population excluding those with no recorded respiratory support post-randomisation. The CEA also excluded patients who did not consent to receive the follow-up HSQ at six months following randomisation.

### Setting, patients and clinical management

FIRST-ABC was a pragmatic, multicentre, randomised, non-inferiority trial conducted across 24 paediatric critical care units (PCCUs) in the United Kingdom. The target population of the trial was critically ill children aged from birth (> 36 weeks corrected gestational age) to 15 years clinically assessed to require NRS for (A) an acute illness (step-up RCT) or (B) following extubation after a period of invasive ventilation (step-down RCT). The study protocol is reported elsewhere [14]. Each RCT (step-up and step-down) recruited 600 patients between August 2019 and November 2021, with last follow-up completed on 18 March 2022. In each RCT, eligible patients were randomised on a 1:1 basis to either CPAP or HFNC, utilizing a dedicated, centralized 24/7 telephone/web-based randomization service.

Children in the active randomized group (HFNC) received HFNC at a prescribed gas flow rate based on patient body weight. Children randomized to CPAP started treatment at an expiratory pressure of 7–8 cm H_2_O. The trial did not specify any particular device or patient interface for the provision of HFNC or CPAP, they were delivered through approved devices and interfaces already in use at sites. For both groups, the fraction of inspired oxygen (FiO_2_) was titrated to maintain peripheral oxygen saturations (SpO_2_) of ≥ 92%. To standardise treatment, clinical criteria and guidance for the initiation, maintenance and weaning of CPAP were provided in the trial. Patients were recommended to be assessed for response to the treatment, readiness to wean and for stopping HFNC/CPAP at least twice per day.

### Health economic outcomes

HrQoL data were collected for surviving trial patients at six months post-randomisation via age-appropriate Paediatric Quality of Life Generic Core Scales (PedsQL^™^) completed by the parents [15]. The responses to PedsQL questionnaire were then mapped onto the Child Health Utility 9D (CHU-9D) index score using an appropriate mapping function [16, 17]. The mapped CHU-9D index score was then combined with survival data to calculate Quality-Adjusted Life Years (QALYs). QALYs at six months post-randomisation were calculated by valuing each patient’s survival time by their HrQoL (as indicated by the mapped CHU-9D utility score) at six months according to the ‘area under the curve’ approach [18]. For six months survivors, QALYs was calculated using the CHU-9D scores at six months, assuming a CHU-9D score of zero at randomisation, and a linear interpolation between randomization and six months. For those who died between randomisation and six months, a zero QALY was assumed for the six-month period. An alternative published algorithm for mapping PedsQL to the CHU-9D index score was considered in a sensitivity analysis (see analysis).

### Resource use and costs

The cost analysis took an NHS and personal social services perspective as recommended by NICE [19], and considered the following resource use categories as the main drivers of incremental costs: critical care and general medical ward length of stay, and use of outpatient and community health services. In the UK healthcare setting, no additional intervention costs were considered necessary for delivering the interventions in paediatric critical care, i.e., Paediatric Intensive Care Unit (PICU)/High Dependency Unit (HDU). Costs incurred by parent, family members of the patient or cost incurred outside the health sector were not included in the cost analysis as they were outside the scope of the chosen perspective of the cost analysis.

The length of stay within PICU/HDU and general medical ward were calculated for both the index admission and any readmissions up to six months. The length of stay within PICU/HDU was costed according to intensity of care provided to each patient each day, the information of which is recorded in the daily interventions recorded in the PICANet database. Each activity day within PICU/HDU was assigned a Healthcare Resource Group (HRG) applying the 2019/20 HRG4 + Grouper algorithm [20]. Patients receiving HFNC or CPAP in a PICU or HDU were considered to receive the same level of care. Intermediate paediatric critical care (XB06Z HRG code) was considered the appropriate level of critical care associated with providing these interventions. The cost of HFNC use in the paediatric ward following discharge from HDU/PICU was costed as basic critical care (XB07Z HRG code). Stay in the paediatric ward not requiring NRS was costed as enhanced care (XB09Z HRG code). Resource utilization stemming from hospital outpatient visits and community services, post-discharge from the initial hospital admission but prior to the six months post-randomization, were sourced from HSQ. Total costs were calculated by combining the resource use with unit costs at 2020/21 prices (£ GBP) (see supplementary material Appendix Table 1).

**Table 1:** Baseline characteristics Step-down RCT

Characteristic	HFNC (n = 254)	CPAP (n = 228)
Age, median (IQR), months	3 (1–10)	4 (1–12)
*Sex, no. (%)*		
Female	97 (38.2)	112 (49.1)
Male	157 (61.8)	116 (50.9)
At least one comorbidity, no. (%)	153 (60.2)	122 (53.5)
*Main reason for invasive ventilation, no. (%)*		
Bronchiolitis	91 (35.8)	111 (48.7)
Other respiratory condition	36 (14.2)	25 (11.0)
Asthma/Wheeze	1 (0.4)	5 (2.2)
Sepsis/infection	9 (3.5)	7 (3.1)
Cardiac	73 (28.7)	39 (17.1)
Upper airway problem	9 (3.5)	11 (4.8)
Neurological	7 (2.8)	13 (5.7)
Other	28 (11.0)	17 (7.5)
Duration of prior invasive ventilation, median (IQR), hour	90 (57–153)	84 (52–137)
Main reason for invasive ventilation, no. (%)		
Planned (randomized before extubation)	160 (63.0)	137 (60.12)
Indeterminate (randomized within 1 h of extubation)	45 (17.7)	46 (20.2)
Rescue (randomized at least 1 h after extubation)	49 (19.3)	45 (19.7)
*Clinical characteristics at randomization*^*a*^		
Respiratory distress, No./total (%)^b^	n = 191	n = 168
None	116 (60.7)	93 (55.4)
Mild	53 (27.8)	46 (27.4)
Moderate	20 (10.5)	24 (14.3)
Severe	2 (1.1)	5 (3.0)
Respiratory rate, median (IQR), [N], breaths per minute	35 (27–44) [250]	35 (27–45) [227]
Peripheral oxygen saturation, median (IQR), [no.], %	96 (94–99) [254]	97 (94–99) [227]
Fraction of inspired oxygen, median (IQR), [no.]	0.30 (0.25–0.35) [232]	0.30 (0.25–0.35) [202]
Ratio of peripheral oxygen saturation to fraction of inspired oxygen, median (IQR), [no.]	325 (271–400) [252]	327 (271–396) [226]
Heart rate, median (IQR), [no.], beats per minute	128 (115–144) [253]	131 (114–147) [228]
COMFORT-B score,^c^ mean (SD), [no.]	13.8 (2.7) [184]	14.2 (3.0) [154]

^a^Data were recorded at or within one hour prior to randomization, except for COMFORT-B score, which was the last recorded value prior to randomization

^b^Respiratory distress was defined as Mild: one accessory muscle used, mild indrawing of subcostal and intercostal muscles, mild tachypnea, no grunting; Moderate: two accessory muscles used, moderate indrawing of subcostal and intercostal muscles, moderate tachypnea, occasional grunting; Severe: use of all accessory muscles, severe indrawing of subcostal and intercostal muscles, severe tachypnea, regular grunting

^c^COMFORT Behaviour (COMFORT-B) scale scores range from 5 to 30 (most sedated to least sedated). A mean value of 15 indicates a comfortable patient who is easily arousable and is not agitated

*RCT* Randomised Controlled Trial, *HFNC* High-flow nasal cannula, *CPAP* Continuous positive airway pressure, *IQR* inter-quartile range

### Analysis

The CEA followed a prespecified analysis plan published before the end of trial recruitment [21]. The CEA followed the mITT principle, and used regression models to report adjusted mean differences between the randomised groups, for life years, HrQoL, QALYs and costs together with 95% confidence intervals (CIs). Incremental life years and HrQoL were estimated using linear regression models. Incremental costs and QALYs were estimated with recommended seemingly unrelated regression (SUR) model to allow for correlation between costs and QALYs, which were subsequently used to calculate the incremental net benefit (INB) [22]. The regression models as per the statistical analysis plan adjusted for residual imbalance in important baseline covariates to increase statistical efficiency [21, 23]. The adjusted baseline variables were: in both RCTs: age (< 12 months versus ≥ 12 months), SpO2:FiO2 ratio at randomisation (linear), co-morbidities (none versus neurological/neuromuscular versus Other); step-up RCT only: severity of respiratory distress at randomisation (severe versus mild/moderate), reason for admission (bronchiolitis versus other respiratory (airway problem, asthma/wheeze or any other respiratory) versus cardiac versus other (neurologic, sepsis/infection, any other), whether the patient was on NRS at randomisation (yes/no); step-down RCT only: length of prior Invasive Mechanical Ventilation (IMV) (< 5 days vs. ≥ 5 days), reason for IMV (cardiac versus other), and nature of noninvasive respiratory support. The primary outcome of CEA is the INB at six months of HFNC versus CPAP. INB was calculated by valuing incremental QALYs at the National Institute for Health and Care Excellence (NICE) recommended threshold of £20,000 per QALY gained, and then subtracting the incremental costs [19]. Missing data in each trial (see supplementary material Appendix Table 2) were addressed with ‘multivariate imputation by chained equations’ (MICE) [24] assuming that the data were missing at random conditional on baseline covariates, resource use and observed endpoints. The resultant estimates were combined with Rubin’s rules, which recognise uncertainty both within and between imputations [25].

The joint uncertainties around estimates of incremental costs and QALYs was represented on a cost-effectiveness plane. We used the estimates of the means, variances and the covariance from the regression model, to randomly generate 800 estimates of incremental costs and QALYs from the joint distribution of these endpoints, assuming asymptotic normality. We have performed pre-specified subgroup analyses and reported INBs for each subgroup. The subgroups were: in both RCTs: age (< 12 months, >  = 12 months), SpO2/FiO2 (SF) ratio at randomisation (quintiles), co-morbidities (none, neurologic/neuromuscular, other); step-up RCT only: severity of respiratory distress at randomisation (not severe, severe), reason for admission (bronchiolitis, other respiratory, cardiac, other), whether the patient was on NRS at randomisation (no, yes); step-down RCT only: length of prior IMV (0–4 days, 5 + days), reason for respiratory support post-extubation, categorised as planned (randomisation followed by extubation), indeterminate (extubation followed by randomisation within 60 min of extubation) vs rescue (extubation followed by randomisation more than 60 min post extubation) breathing support, and reason for IMV (not cardiac, cardiac). Subgroup effects were estimated by treatment interactions.

We undertook sensitivity analyses to investigate the impact of making different assumptions to the base case INB results (see supplementary material Appendix Table 4). In the sensitivity analyses we varied (a) intention to treat analysis sample (rather than mITT sample), (b) intervention based costing of interventions (rather than location based), (c) follow-up costs from HES databases (rather than HSQ), (d) ± 10% increase/decrease in unit costs, (e) CHU-9D mapping algorithm from Australian population (rather than UK population), (f) complete case analysis, (g) Gamma rather than a Normal distribution for costs and QALYs in regression model, and (h) multilevel regression model to allow for clustering of patients at sites rather than single level regression model.

### Ethical approval

The FIRST-ABC master protocol was approved by the East of England–Cambridge South Research Ethics Committee (19/EE/0185) and the UK Health Research Authority (IRAS 260536). The trial was registered with the International Standard Randomised Controlled Trial Number (ISRCTN) Registry (ISRCTN60048867).

## Results

### Patient characteristics

The economic analysis included 482 trial participants (HFNC 254, CPAP 228) for the step-down RCT and 506 trial participants (HFNC 269, CPAP 237) for the step-up RCT, representing 87% and 88% of the participants included in the clinical effectiveness analyses respectively. The randomised groups in the economic analysis had similar baseline characteristics to both RCTs (Tables 1 and 2). There were some baseline imbalances in sex, proportion of at least one comorbidity, main reason for invasive ventilation, and duration of prior invasive ventilation in the step-down trial. In the step-up trial, there were some imbalances in main reason for admission and ratio of peripheral oxygen saturation to fraction of inspired oxygen.

**Table 2:** Baseline characteristics Step-up RCT

Characteristic	HFNC (N = 269)	CPAP (N = 237)
Age, median (IQR), months	9 (2–30)	9 (1–26)
*Sex, no. (%)*		
Female	108 (40.1)	92 (38.8)
Male	161 (59.9)	145 (61.2)
At least one comorbidity, no. (%)	n = 269	n = 236
	130 (48.3)	104 (44.1)
*Main reason for admission, no. (%)*	n = 269	n = 236
Bronchiolitis	131 (48.7)	121 (51.3)
Other respiratory condition	54 (20.1)	45 (19.1)
Asthma/Wheeze	29 (10.8)	20 (8.5)
Sepsis/infection	20 (7.4)	18 (7.6)
Cardiac	16 (6.0)	11 (4.7)
Upper airway problem	11 (4.1)	10 (4.2)
Neurological	3 (1.1)	2 (0.9)
Other	5 (1.9)	9 (3.8)
*On non-invasive respiratory support at randomization, no. (%)*		
No	205 (76.2)	181 (76.4)
Yes	64 (23.8)	56 (23.6)
*Clinical characteristics at randomization*^*a*^		
Respiratory distress, no./N (%)^b^	n = 225	n = 194
None	11 (4.9)	9 (4.9)
Mild	43 (19.1)	32 (16.5)
Moderate	129 (57.3)	121 (62.4)
Severe	42 (18.7)	32 (16.5)
Respiratory rate, median (IQR), [No.], breaths per minute	48 (38–60) [261]	49 (40–60) [233]
Peripheral oxygen saturation, median (IQR), [No.], percentage	97 (94–99) [265]	97 (94–99) [236]
Fraction of inspired oxygen, median (IQR), [No.]	0.32 (0.21–0.50) [232]	0.28 (0.21–0.44) [232]
Ratio of peripheral oxygen saturation to fraction of inspired oxygen, median (IQR), [No.]	285 (194–417) [232]	331 (217–443) [232]
Heart rate, median (IQR), [No.], beats per minute	154 (139–171) [233]	156 (140–173) [233]
COMFORT-B score,^c^ mean (SD), [No.]	16.2 (4.8) [77]	15.3 (5.5) [56]

^a^Data were recorded at or within one hour prior to randomization, except for COMFORT-B score, which was the last recorded value prior to randomization

^b^Respiratory distress was defined as Mild: one accessory muscle used, mild indrawing of subcostal and intercostal muscles, mild tachypnea, no grunting; Moderate: two accessory muscles used, moderate indrawing of subcostal and intercostal muscles, moderate tachypnea, occasional grunting; Severe: use of all accessory muscles, severe indrawing of subcostal and intercostal muscles, severe tachypnea, regular grunting

^c^COMFORT Behaviour (COMFORT-B) scale scores range from 5 to 30 (most sedated to least sedated). A mean value of 15 indicates a comfortable patient who is easily arousable and is not agitated

RCT Randomised Controlled Trial, *HFNC* High-flow nasal cannula, *CPAP* Continuous positive airway pressure, *IQR* inter-quartile range

### Health outcomes

In the step-down RCT, all-cause mortality rates within six months were low in both randomised groups, although it was slightly higher in the HFNC compared to CPAP group, resulting in slightly lower mean life years in the HFNC compared to the CPAP group (Table 3). Among patients who were alive at six months, the lowest PedsQL scores were in the emotional domain, and the highest in the social domain (see Supplementary Table 4). Mean total PedsQL score and mean mapped CHU-9D score of patients who were alive at six months were similar between the randomised groups (see supplementary material Appendix Tables 3 and 4). Mean QALYs at six months were 0.218 in the HFNC group and 0.227 in the CPAP group.

**Table 3:** Mortality, Child Health Utility 9 Dimension (CHU9D) score, life years, and quality-adjusted life years (QALY) up to six months

	Step-down RCT		Step-up RCT	
	HFNC (n = 254)	CPAP (n = 228)	HFNC (n = 269)	CPAP (n = 237)
All-cause mortality: n (%)	13 (5.20)	2 (0.89)	9 (3.35)	3 (1.27)
CHU9D utility score (survivors) *	0.920 (0.045)	0.919 (0.053)	0.919 (0.033)	0.917 (0.036)
Life years*	0.473 (0.086)	0.490 (0.030)	0.483 (0.090)	0.494 (0.056)
QALY*	0.218 (0.052)	0.227 (0.027)	0.222 (0.042)	0.226 (0.027)

Mean (SD) reported unless stated otherwise

*RCT* Randomised Controlled Trial, *HFNC* High-flow nasal cannula, *CPAP* Continuous positive airway pressure, *CHU9D* Child Health Utility 9 Dimension, *QALY* Quality-Adjusted Life Years, SD standard deviation

*Following multiple imputation to handle missing data

**Table 4:** Resource use and costs up to six months. Mean (SD) reported unless stated otherwise

	Step-down RCT		Step-up RCT	
	HFNC (n = 254)	CPAP (n = 228)	HFNC (n = 269)	CPAP (n = 237)
(a) Resource use				
*Hospital stay (days)*				
Index admission: PICU/HDU	7.43 (13.73)	7.88 (16.38)	5.08 (11.04)	7.05 (17.40)
Index admission: general medical ward	11.63 (23.77)	10.72 (22.86)	9.04 (25.41)	9.74 (26.71)
Readmission^a^: PICU/HDU	0.65 (3.08)	1.32 (8.85)	1.15 (6.31)	1.24 (7.59)
Readmission^a^: General medical ward	7.11 (12.11)	8.57 (17.06)	4.45 (7.77)	3.65 (8.41)
Total hospital length of stay up to 6 months	26.82 (33.08)	28.49 (37.30)	19.71 (31.06)	21.68 (38.76)
Visits to primary, outpatient & community health services providers (number of visits)^b^	21.03 (20.19)	22.29 (23.75)	9.72 (17.98)	9.65 (19.10)
(b) Costs (2019 £ GB)				
*Hospital stay (costs)*				
Index admission: PICU/HDU	11,755 (24,766)	12,592 (30,808)	8658 (13,738)	12,584 (28,819)
Index admission: general medical ward	9352 (19,112)	8799 (18,706)	6142 (17,115)	6617 (18,111)
Readmission^a^: PICU/HDU	735 (4951)	1335 (11,342)	2126 (10,951)	2134 (12756)
Readmission^a^: General medical ward	5449 (9273)	6565 (13,068)	2990 (5221)	2452 (5653)
Total hospital costs up to 6 months^a^:	27,292 (36,546)	29,291 (42,528)	19,918 (27,130)	23,787 (42,862)
Primary, outpatient & community health services costs*	984 (897)	1013 (1012)	418 (437)	355 (695)
Total costs up to six months*^a,b^	28,275 (36,668)	30,303 (42,710)	20,335 (27,207)	24,142 (42,938)

^a^FIRST-ABC Study and PICANET Database

^b^Patient who were alive and completed the Health Services Questionnaire at six months

*RCT* Randomised Controlled Trial, *HFNC* High-flow nasal cannula, *CPAP* Continuous positive airway pressure, *HFNC* High-flow nasal cannula, *PICU* paediatric intensive care unit, *HDU* high dependency unit, *SD* standard deviation

*Following multiple imputation to handle missing data

Health outcomes in the step-up RCT were similar to that of the step-down RCT. All-cause mortality, mean life years and mean QALYs were similar across randomised groups at six months. In the step-up RCT, mean PedsQL score (for each dimension and total score) and mapped CHU-9D score of patients who were alive at six months were similar between the randomised groups. Mean QALYs at six months were 0.222 in the HFNC group and 0.226 in the CPAP group.

### Resource use and costs

In the step-down RCT, mean paediatric critical care unit length of stay from index hospital admission was about 8 days in both groups (Table 4). Mean length of stay in general medical wards from index hospital admission were about 11–12 days in both groups. From hospital readmissions, mean length of stay in general medical wards compared to critical care unit were higher in both groups. The mean total length of hospital stay at six months was 26.82 days in HFNC group and 28.49 days in CPAP group. On average, total number of visits to primary, outpatient and community health services were similar between the randomised groups in both RCTs (see Table 4 and supplementary material Appendix Table 5).

**Table 5:** Cost-effectiveness at six months: total costs, Child Health Utility 9 Dimension (CHU9D) score, life years, quality-adjusted life years (QALY)

	Step-down RCT			Step-up RCT		
	HFNC (n = 254)	CPAP (n = 228)	Incremental effect^a^ mean (95% CI)	HFNC (n = 269)	CPAP (n = 237)	Incremental effect^a^ mean (95% CI)
Costs (2019 £ GB)*	28,275 (36,668)	30,303 (42,710)	− 4565 (− 11,499– 2368)	20,335 (27,207)	24,142 (42,938)	− 5702 (− 11,328–− 75)
CHU-9D utility score (survivors) *	0.920 (0.045)	0.919 (0.053)	0.002 (− 0.011–0.015)	0.919 (0.033)	0.917 (0.036)	0.002 (− 0.011–0.016)
Life years*	0.473 (0.086)	0.490 (0.030)	− 0.017 (− 0.029–− 0.005)	0.483 (0.090)	0.494 (0.056)	− 0.009 (− 0.022–0.004)
QALY*	0.218 (0.052)	0.227 (0.027)	− 0.009 (− 0.017–− 0.001)	0.222 (0.042)	0.226 (0.027)	− 0.003 (− 0.010–0.004)
INB (2019 £ GB)^b^	4388 (− 2551–11,327)			5628 (− 8–11,264)		

^a^The incremental effects are reported after applying case-mix adjustment

^b^The INB is calculated according to NICE methods guidance, by multiplying the mean QALY gain (or loss) by £20,000, and subtracting from this the incremental cost

Mean (SD) reported unless stated otherwise

*RCT* Randomised Controlled Trial, *HFNC* High-flow nasal cannula, *CPAP* Continuous positive airway pressure, *CHU9D* Child Health Utility 9 Dimension, *QALY* Quality-Adjusted Life Years

*Following multiple imputation to handle missing data

In the step-up RCT, mean length of stay in paediatric critical care units from index hospital admission were 5 days in HFNC group and 7 days in CPAP group. Mean length of stay in general medical wards from index hospital admission were about 9–10 days across randomised groups. From hospital readmissions, mean length of stay in general medical wards compared to critical care unit were higher in both groups. The mean total length of hospital stay at six months was 19.71 days in HFNC group and 21.68 days in CPAP group.

In both RCTs, the cost of index hospital stays accounted for a major share of total costs (75% and 71% of total cost up to 6 months in HFNC and CPAP, respectively, in step-down trial, and 73% and 80% in the HFNC and CPAP group, respectively, in step-up trial) for both randomised groups. Within the index admission, costs of stays in the PICUs were higher than that of general medical ward costs (PICU accounts for 55% and 59% of total index admission costs in HFNC and CPAP group, respectively, in step-down trial, and 59% and 65% in the step-up trial). Mean cost per patient in the step-down RCT were £28,275 in the HFNC group and £30,303 in the CPAP group. The corresponding mean costs per patient in the step-up RCT were £19,918 in the HFNC group and £23,787 in the CPAP group.

### Cost-effectiveness

For the step-down RCT, the differences in mean cost after case-mix adjustment at six months was − £4565 surrounded by wide uncertainty (95% CI − £11,499 to £2368) (Table 5). Case-mix adjusted mean incremental HrQoL (mapped CHU-9D score) among the six months survivors, life years and QALYs were small and statistically insignificant.

For the step-up RCT, the differences in mean cost after case-mix adjustment at six months was − £5702 (95% CI − £11,328 to − £75), showing that HFNC is cost saving. The adjusted differences in HrQoL (mapped CHU-9D score) among the six months survivors, life years and QALYs were small and statistically insignificant.

**Fig. 1:**
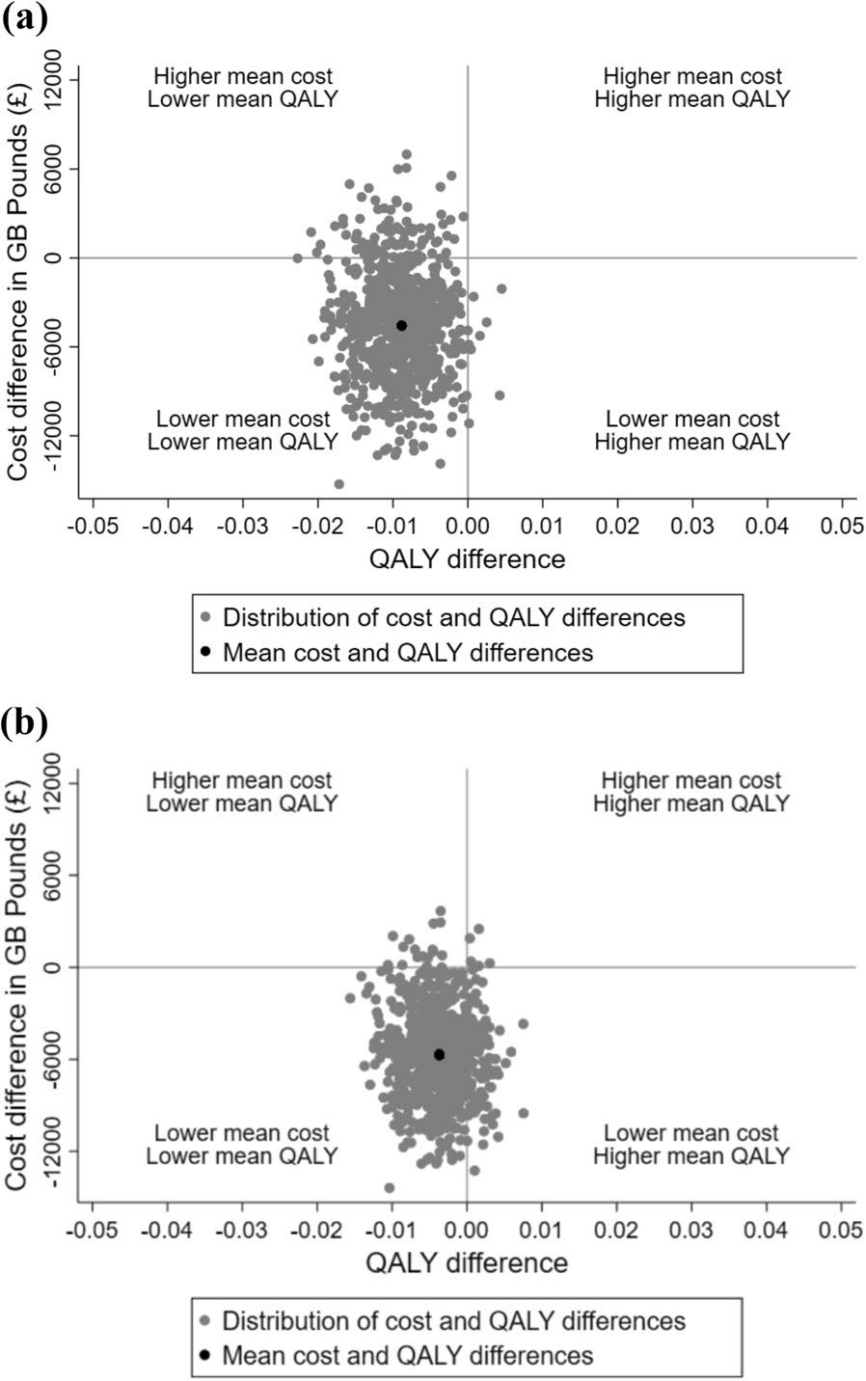
Uncertainty in the total cost and quality-adjusted life years (QALY) differences and their distribution for high-flow nasal cannula (HFNC) versus continuous positive airway pressure (CPAP) within six months post randomisation in Step-down RCT (panel A) and Step-up RCT (panel B)

The incremental results for both RCTs presented on the cost-effectiveness plane shows that the majority of the points were in that quadrant where HFNC had lower QALYs and lower costs (Fig. 1). Hence, the resulting mean INB of HFNC versus CPAP was positive in both RCTs (step-down RCT £4388, step-up RCT £5628), but the statistical uncertainty surrounding this result was substantial (step-down RCT: 95% CI − £2551 to £11,327; step-up RCT: 95% CI − £8 to £11,264). The cost-effectiveness results were similar across most pre-specified sub-groups, except for a few subgroups such as cardiac patients, other comorbidities and SF ratio (in both RCTs). The base case results were robust to alternative scenarios considered in sensitivity analyses (see supplementary material Appendix Figs. 1 and 2).

## Discussion

The main finding of the CEA is that mean costs and QALYs are similar between HFNC and CPAP group in the step-down RCT. In the step-up RCT, HFNC compared to CPAP reduces mean costs but mean QALYs were similar between the randomised groups. This leads to positive INB, albeit with considerable uncertainty surrounding the cost-effectiveness results in both RCTs. The main driver of total costs was index hospital stay accounting for more than 70% of total costs in both randomised groups across the RCTs. Within the index admission, costs of stays in paediatric critical care units were higher than that of general medical ward costs—PICU/HDU stay accounted for more than 55% of total index admission costs across randomised groups and RCTs. The results of the CEA should be interpreted carefully, considering the similar QALY and uncertainty around the incremental costs results. The CEA results for most pre-specified subgroups are similar to the overall results. The sensitivity analysis found that this conclusion is robust to alternative assumptions to those made in the base-case analysis.

Despite increasing adoption of HFNC in clinical practice, there is little evidence demonstrating whether it is cost-effective compared to CPAP [26]. This study adds to the scarce evidence on the cost-effectiveness of HFNC versus CPAP as first line NRS in the paediatric critical care population. Previous studies had found that HFNC is likely to be cost-effective compared to standard oxygen therapy (low-flow) [27, 28] whereas one other study found that HFNC could not be deemed cost-saving [29]. One recent decision tree-based cost-utility analysis suggested that HFNC is less cost-effective than CPAP for treating children < 2 years old with acute and moderate or severe bronchiolitis [30]. However, the study does not use data from head-to-head trials. Economic evaluation conducted alongside the HIPSTER trial reported that HFNC with rescue CPAP is likely to be cost-effective compared to CPAP for treating respiratory distress syndrome in infants born preterm [31]. However, it is unclear whether these findings might be generalisable to the population of critically ill children. Our study addresses the gap in evidence in the literature and reported cost-effectiveness alongside the first ever RCTs comparing two commonly used modes of NRS in critically ill children. We found that HFNC was associated with considerably reduced costs for step-up and step-down RCTs and HrQoL was similar between the randomised groups. However, mortality was relatively higher for the HFNC group leading to lower mean QALY in HFNC group, the reason for which is not clear. Possible explanations include a difference in baseline characteristics between the groups (higher proportion of cardiac patients in the HFNC group in the step-down RCT for example) and clinical deterioration associated with earlier discharge to ward in HFNC patients (most HFNC deaths occurred between PICU discharge and hospital discharge).

This study has several notable strengths. Firstly, it is the first large-scale report of the cost-effectiveness of NRS in children. We employed a prospectively designed economic evaluation integrated with well-designed step-up and step-down RCTs, and collected detailed patient level resource use and health economic outcome data from multiple linked databases. Secondly, HrQoL in the study was measured with widely used age-appropriate PedsQL instruments for children and adolescents, which were then mapped to a preference based CHU-9D utility score for calculating QALYs, which is suitable and preferred outcome measure for economic evaluation. Thirdly, the economic analysis adhered to a harmonized and pre-specified statistical and economic analysis plan, with comprehensive sensitivity analyses, and showed that the base-case results were not sensitive to alternative assumptions.

The study was subject to a few limitations. We measured HrQoL using CHU-9D, which is not validated in younger children. We have therefore calculated mapped CHU-9D score from PedsQL. We used alternative mapping algorithm for mapping and found that the cost-effectiveness results were not sensitive to alternative mapping algorithm. The CEA followed the mITT principle as undertaken in the analysis of primary clinical outcome of FIRST-ABC RCTs and excluded patients due to non-consent to the follow-up questionnaire, which could potentially weaken the inferences from the economic analysis. In the FIRST-ABC RCTs, 25–45% of children crossed over from the initial mode of NRS to other modes. Therefore, we have also carried out sensitivity analysis on the ITT population, and the results were similar to that of the mITT population. There were some missing data for the resource use items and HrQoL collected through the HSQ, which were imputed using the recommended MICE approach. Among the patients included for economic analysis there were some differences in baseline characteristics across the randomised groups, which could bias the study findings. The trial recruitment was primarily of younger children under 12 months of age although the eligible trial population was children aged 0–15 years. The results of the study therefore may not be generalisable to the entire paediatric population.

The CEA presented results for the same pre-specified subgroups as for the analysis of clinical effectiveness. The cost-effectiveness results were consistent across all subgroups. The results of these subgroup analysis suggested that the point estimate of the INB was positive for some subgroups (such as cardiac patients, other comorbidities and SF ratio), negative for others, but that the CIs around each of these estimates were wide and included zero. When interpreting these findings, it is essential to recognise that this study was not designed to detect a subgroup effect for either clinical effectiveness or cost-effectiveness endpoints; hence, the subgroup results should be viewed as exploratory.

The results from the pre-specified subgroup analyses pose the hypothesis that some subgroups (for instance, the cardiac patient subgroup in both step-up and step-down RCTs) may find the HFNC intervention more cost-effective than CPAP. Further research could assess the clinical and cost-effectiveness of targeted interventions for subgroups admitted to paediatric critical care units for cardiac reasons.

## Conclusion

HFNC reduces mean costs and is likely to be cost-effective.

## Supplementary Information

Additional file1 (DOCX 1803 kb)

## Data Availability

All data are presented in tables and figures. Any supplementary data are available from the corresponding author on reasonable request.

## Abbreviations

CEA: Cost-effectiveness analysis
CHU-9D: Child health utility 9D
CPAP: Continuous positive airway pressure
CRF: Case report form
FiO_2_: Fraction of inspired oxygen
FIRST-ABC: FIRST-line support for assistance in breathing in children
HDU: High dependency unit
HES: Hospital episode statistics
HFNC: High flow nasal cannula therapy
HRG: Healthcare resource group
HrQoL: Health-related quality of life
IMV: Invasive mechanical ventilation
INB: Incremental net benefit
IQR: Inter-quartile range
ITT: Intention to treat
MICE: Multivariate imputation by chained equations
mITT: Modified intention-to-treat principle
NRS: Non-invasive respiratory support
PedsQL: Paediatric quality of life generic core scales
PICANet: Paediatric intensive care audit network
PICU: Paediatric intensive care unit
PCCU: Paediatric critical care unit
RCT: Randomised controlled trial
SD: Standard deviation
SF: SpO_2_/FiO_2_
SpO_2_: Peripheral oxygen saturations
QALY: Quality-adjusted life year
