# Supplementary material for: Cost-effectiveness of high flow nasal cannula therapy versus continuous positive airway pressure for non-invasive respiratory support in paediatric critical care
Source: Crit Care. 2024 Nov 25;28:386. doi: 10.1186/s13054-024-05148-y (PMC11587665; doi:10.1186/s13054-024-05148-y)

**Appendix Table 1. Unit costs (£)**

| Items | Unit costs | Source |
| --- | --- | --- |
| Hospital costs (bed day) |  |  |
| Enhanced Care | 766 | NHS National Benchmark price |
| Basic Critical Care | 1,149 | NHS National Benchmark price |
| Intermediate Critical Care | 1,437 | NHS National Benchmark price |
| Advanced Critical Care 1 | 1,916 | NHS National Benchmark price |
| Advanced Critical Care 2 | 2,395 | NHS National Benchmark price |
| Advanced Critical Care 3 | 2,874 | NHS National Benchmark price |
| Advanced Critical Care 4 | 3,832 | NHS National Benchmark price |
| Advanced Critical Care 5 | 5,747 | NHS National Benchmark price |
| General medical bed day | 766 | NHS National Benchmark price |
| Outpatient & community health services |  |  |
| Hospital outpatient | 135 | PSSRU |
| GP practice visit (per visit) | 39 | PSSRU |
| GP home visit (per visit) | 90 | PSSRU |
| GP nurse visit† | 11 | PSSRU |
| GP nurse home visit† | 19 | PSSRU |
| Hospital nurse† | 10 | PSSRU |
| Health visitor† | 8 | PSSRU |
| Health visitor home visit† | 14 | PSSRU |
| Occupational therapist† | 9 | PSSRU |
| Physiotherapist† | 9 | PSSRU |
| Psychiatrist† | 29 | PSSRU |
| Paediatric nurse† | 9 | PSSRU |
| School nurse† | 12 | PSSRU |
| Social worker† | 13 | PSSRU |
| Counsellor† | 9 | PSSRU |
| Speech and language therapist† | 9 | PSSRU |
| Dietician† | 9 | PSSRU |
| Midwife† | 9 | PSSRU |

* PSSRU: Personal Social Services Research Unit; NHS: National Health Service.

†15 minutes of consultation time.

**Appendix Table 2(a). Step-up RCT - Variables considered for multiple imputation and form of imputation model**

| **Variable** | **Missing values,**  **n (%)** | **Imputation model** |
| --- | --- | --- |
| **Baseline covariates** |  |  |
| Allocated treatment group | 0 (0) | None required |
| Age | 0 (0) | None required |
| On support at randomisation | 0 (0) | None required |
| Main reason for admission | 1 (0.20) | Multinomial logit |
| Comorbidities | 1 (0.20) | Multinomial logistic regression |
| SpO2/FiO2 ratio | 12 (2.37) | Predictive mean matching |
| Severe respiratory distress at baseline | 87 (17.19) | Logistic regression |
| **Resource use** |  |  |
| Index hospital admission |  |  |
| Duration of stay in the PICU/HDU | 0 (0) | None required |
| Duration of stay in general medical ward | 0 (0) | None required |
| Readmissions up to six months |  |  |
| Duration of stay in the PICU/HDU | 0 (0) | None required |
| Duration of stay in general medical ward | 315 (62.25) | Predictive mean matching |
| **Outcomes** |  |  |
| Mortality | 3 (0.59) | Logistic regression |
| CHU9D utility score | 344 (67.98) | Predictive mean matching |
| Health services questionnaire costs | 315 (62.25) | Predictive mean matching |

PICU: paediatric intensive care unit, HDU: high dependency unit, CHU-9D: Child Health Utility questionnaire.

**Appendix Table 2(b). Step-down RCT - Variables considered for multiple imputation and form of imputation model**

| **Variable** | **Missing values,**  **n (%)** | **Imputation model** |
| --- | --- | --- |
| **Baseline covariates** |  |  |
| Allocated treatment group | 0 (0) | None required |
| Age | 0 (0) | None required |
| Reason for IMV | 0 (0) | None required |
| Duration of prior IMV | 0 (0) | None required |
| Comorbidities | 0 (0) | None required |
| Planned respiratory support | 0 (0) | None required |
| SpO2/FiO2 ratio | 4 (<0.1) | Predictive mean matching |
| **Resource use** |  |  |
| Index hospital admission |  |  |
| Duration of stay in the PICU/HDU | 0 (0) | None required |
| Duration of stay in general medical ward | 0 (0) | None required |
| Readmissions up to six months |  |  |
| Duration of stay in the PICU/HDU | 0 (0) | None required |
| Duration of stay in general medical ward | 158 (34.4) | Predictive mean matching |
| **Outcomes** |  |  |
| Mortality | 8 (1.7) | Logistic regression |
| CHU9D utility score | 270 (58.8) | Predictive mean matching |
| Health services questionnaire costs | 230 (50.1) | Predictive mean matching |

IMV invasive mechanical ventilation, PICU: paediatric intensive care unit, HDU: high dependency unit, CHU-9D: Child Health Utility questionnaire.

**Appendix Table 3. Alternative assumptions for cost-effectiveness sensitivity analyses for both step-up and step-down trial**

|  | **Base case** | **Sensitivity analysis** |
| --- | --- | --- |
| Analysis sample | Modified intention to treat (mITT) | Intention to treat (ITT) |
| Intervention costs | Location based | Intervention based |
| Follow-up costs | Health Services Questionnaires | Hospital Episode Statistics database |
| Unit costs of resources | NHS Benchmark prices and PSSRU costs in 2021/21 | +/- 10% increase/decrease in all unit costs |
| CHU9D mapping | Mapping algorithm from UK population applied | Mapping algorithm from Australian population applied |
| Analysis model and missing data | Full cohort using MI methods | Complete cases only |
| Distributional assumptions | Costs and QALYs normally distributed | Costs and QALYs gamma distributed |
| Modelling assumption | Single level bivariate regression model | Multilevel bivariate regression model to allow for clustering of patients at sites. |

CHU9D Child Health Utility 9 Dimension; QALY Quality-Adjusted Life Years.

**Appendix Table 4(a). Step-down RCT - PedsQL score at six months, Mean (SD)**

|  | **HFNC**  **(n=114)** | **CPAP**  **(n=75)** |
| --- | --- | --- |
| **PedsQL dimension** |  |  |
| Physical score* | 79 (14) | 81 (16) |
| Emotional score* | 71 (18) | 74 (17) |
| Social score* | 89 (15) | 89 (18) |
| School score* | 79 (22) | 77 (23) |
| **PedsQL total score*** | 77 (13) | 79 (14) |

* Only reported for patients who were alive and completed the follow-up questionnaires at six months post-randomisation

**Appendix Table 4(b). Step-up RCT - PedsQL score at six months, Mean (SD)**

|  | **HFNC**  **(n=269)** | **CPAP**  **(n=237)** |
| --- | --- | --- |
| **PedsQL dimension** |  |  |
| Physical score* | 70 (31) | 73 (28) |
| Emotional score* | 71 (18) | 68 (20) |
| Social score* | 81 (20) | 81 (23) |
| School score* | 65 (28) | 67 (30) |
| **PedsQL total score*** | 71 (21) | 72 (22) |

* Only reported for patients who were alive and completed the follow-up questionnaires at six months post-randomisation.

**Appendix Table 5(a). Step-down RCT – Mean (SD) resource use from Health Services Questionnaire between discharge from hospital and six months following initial critical care episode for patients who were alive and completed the questionnaire at six months post randomisation**

|  | **HFNC**  **(n=168)** | **CPAP**  **(n=133)** |
| --- | --- | --- |
| Outpatient visits | 5.42 (6.14) | 5.15 (6.26) |
| GP contacts | 3.06 (3.58) | 2.77 (3.54) |
| Nurse contacts | 1.75 (2.33) | 1.58 (2.04) |
| Health visitor contacts | 6.64 (7.94) | 6.72 (9.23) |
| Counsellor contacts | 0.16 (0.40) | 0.15 (0.36) |
| Dietician contacts | 2.62 (4.10) | 2.77 (4.29) |
| Midwife visits | 0.27 (0.65) | 0.26 (0.64) |
| Occupational therapist contacts | 0.70 (1.95) | 1.33 (2.52) |
| Psychiatric nurse contacts | 0.23 (0.56) | 0.24 (0.66) |
| Physiotherapist contacts | 1.83 (3.86) | 3.14 (4.93) |
| School nurse visits | 0.28 (0.78) | 0.19 (0.49) |
| Social worker visits | 0.46 (1.40) | 0.85 (2.12) |
| Paediatric nurse visits | 2.09 (4.19) | 1.98 (4.11) |
| Speech therapist contacts | 1.88 (3.43) | 1.63 (2.9) |

**Table 5(b). Step-up RCT - Mean (SD) resource use from Health Services Questionnaire between discharge from hospital and six months following initial critical care episode for patients who were alive and completed the questionnaire at six months post randomisation**

|  | **HFNC**  **(n=99)** | **CPAP**  **(n=81)** |
| --- | --- | --- |
| Outpatient visits | 2.37 (4.10) | 2.75 (7.85) |
| GP contacts | 1.61 (3.00) | 1.14 (1.66) |
| Nurse contacts | 0.47 (1.64) | 0.30 (0.97) |
| Health visitor contacts | 2.59 (7.47) | 1.91 (5.48) |
| Counsellor contacts | 0.20 (2.02) | 0.00 (0.00) |
| Dietician contacts | 0.33 (1.00) | 0.70 (2.75) |
| Midwife visits | 0.00 (0.00) | 0.00 (0.00) |
| Occupational therapist contacts | 0.31 (1.30) | 0.65 (3.40) |
| Psychiatric nurse contacts | 0.08 (0.55) | 0.22 (1.89) |
| Physiotherapist contacts | 0.69 (2.47) | 1.20 (2.72) |
| School nurse visits | 0.03 (0.17) | 0.09 (0.60) |
| Social worker visits | 0.04 (0.25) | 0.14 (0.82) |
| Paediatric nurse visits | 0.70 (2.45) | 0.33 (1.75) |
| Speech therapist contacts | 0.30 (1.34) | 0.22 (0.79) |

* Only reported for patients who were alive and completed the HSQ at six months post-randomisation.

**Appendix Figure 1(a). Step-down RCT - Sub-group analysis for the cost-effectiveness analysis at six months**


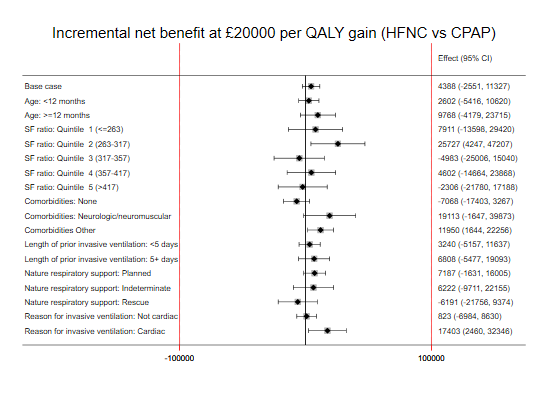


**Appendix Figure 1(b). Step-up RCT - Sub-group analysis for the cost-effectiveness analysis at six months**


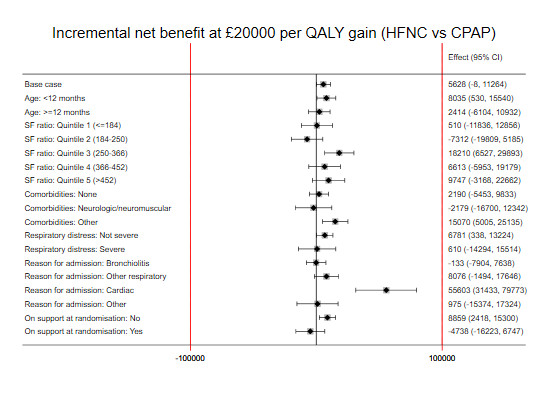


**Appendix Figure 2(a). Step-down RCT - Sensitivity analysis for the cost-effectiveness analysis at six months**


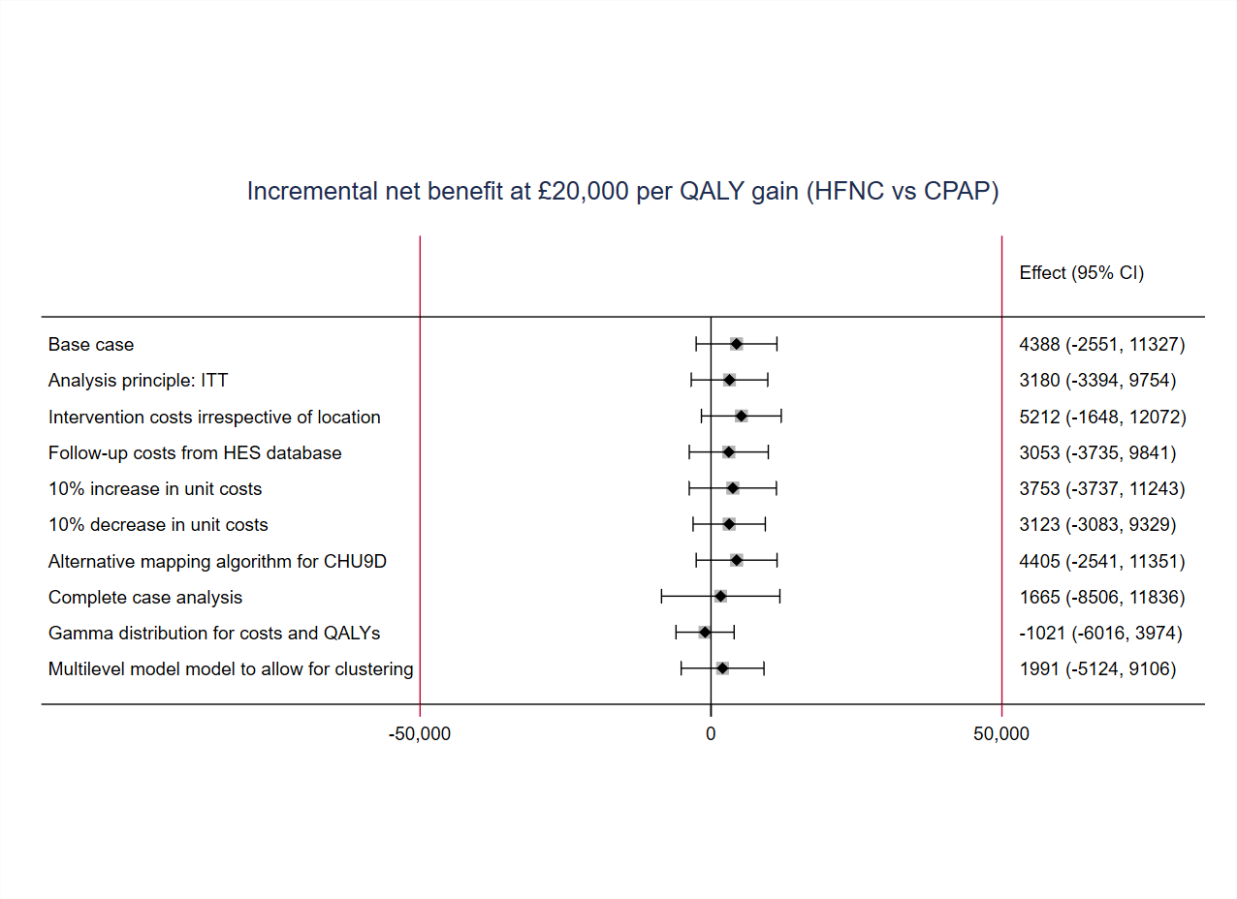


**Appendix Figure 2(b). Step-up RCT - Sensitivity analysis for the cost-effectiveness analysis at six months**


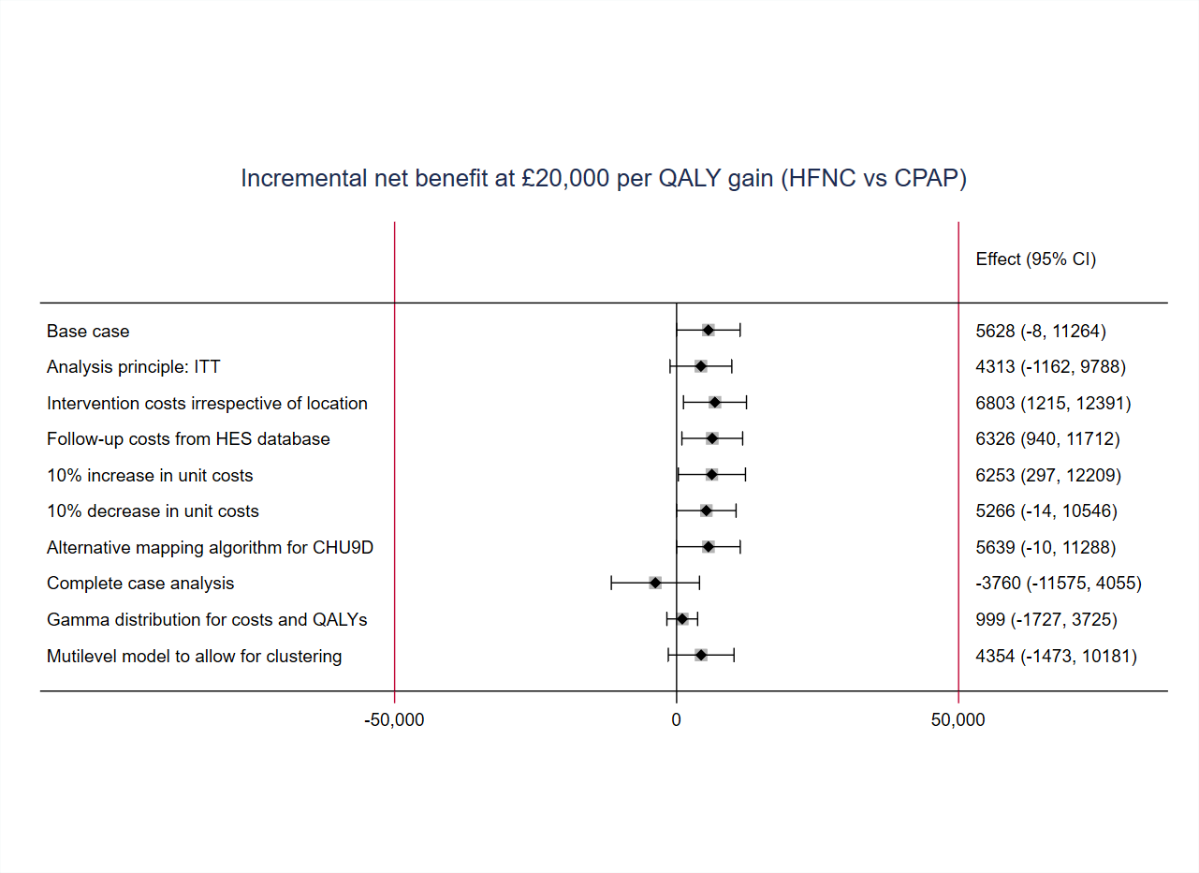

Supplement: Supplementary file 1 — Additional file1 (DOCX 1803 kb) [file 13054_2024_5148_MOESM1_ESM.docx]
